# Andrographolide Ameliorates Inflammation and Fibrogenesis and Attenuates Inflammasome Activation in Experimental Non-Alcoholic Steatohepatitis

**DOI:** 10.1038/s41598-017-03675-z

**Published:** 2017-06-14

**Authors:** Daniel Cabrera, Alexander Wree, Davide Povero, Nancy Solís, Alejandra Hernandez, Margarita Pizarro, Han Moshage, Javiera Torres, Ariel E. Feldstein, Claudio Cabello-Verrugio, Enrique Brandan, Francisco Barrera, Juan Pablo Arab, Marco Arrese

**Affiliations:** 1 Departamento de Gastroenterología, Escuela de Medicina, Pontificia Universidad Católica de Chile, Santiago, Chile; 2 Departamento de Ciencias Químicas y Biológicas, Facultad de Salud, Universidad Bernardo O Higgins, Santiago, Chile; 3 Department of Pediatrics, University of California, San Diego, USA; 4 Department of Gastroenterology and Hepatology, University Medical Center Groningen, University of Groningen, Groningen, Netherlands; 5 Departamento de Patología, Escuela de Medicina, Pontificia Universidad Católica de Chile, Santiago, Chile; 6 Laboratorio de Biología y Fisiopatología Molecular, Departamento de Ciencias Biológicas, Facultad de Ciencias Biológicas & Facultad de Medicina, Universidad Andrés Bello, Santiago, Chile; 7 Millennium Institute on Immunology and Immunotherapy, Santiago, Chile; 8 Departamento de Biología Celular y Molecular, Facultad de Ciencias Biológicas, Pontificia Universidad Católica de Chile, Santiago, Chile; 9 Centro de Envejecimiento y Regeneración (CARE), Departamento de Biología Celular y Molecular, Facultad de Ciencias Biológicas Pontificia Universidad Católica de Chile, Santiago, Chile; 10 Division of Gastroenterology and Hepatology, Mayo Clinic, Rochester, USA

## Abstract

Therapy for nonalcoholic steatohepatitis (NASH) is limited. Andrographolide (ANDRO), a botanical compound, has a potent anti-inflammatory activity due to its ability to inhibit NF-κB. ANDRO has been also shown to inhibit the NLRP3 inflammasome, a relevant pathway in NASH. Our aim was to evaluate the effects of ANDRO in NASH and its influence on inflammasome activation in this setting. Thus, mice were fed a choline-deficient-amino-acid–defined (CDAA) diet with/without concomitant ANDRO administration (1 mg/kg, 3-times/week). Also, we assessed serum levels of alanine-aminotransferase (ALT), liver histology, hepatic triglyceride content (HTC) and hepatic expression of pro-inflammatory, pro-fibrotic and inflammasome genes. Inflammasome activation was also evaluated in fat-laden HepG2 cells. Our results showed that ANDRO administration decreased HTC and attenuated hepatic inflammation and fibrosis in CDAA-fed mice. ANDRO treatment determined a strong reduction in hepatic macrophage infiltration and reduced hepatic mRNA levels of both pro-inflammatory and pro-fibrotic genes. In addition, mice treated with ANDRO showed reduced expression of inflammasome genes. Finally, ANDRO inhibited LPS-induced interleukin-1β expression through NF-κB inhibition in fat-laden HepG2 cells and inflammasome disassembly. In conclusion, ANDRO administration reduces inflammation and fibrosis in experimental NASH. Inflammasome modulation by a NF-κB-dependent mechanism may be involved in the therapeutic effects of ANDRO.

## Introduction

Nonalcoholic fatty liver disease (NAFLD) comprises a spectrum of liver histological alterations ranging from non-inflammatory isolated steatosis to nonalcoholic steatohepatitis (NASH), which is characterized by steatosis, necro-inflammatory changes and varying degrees of liver fibrosis^1, 2^. NAFLD is considered as a burgeoning health problem currently being the most common causes of chronic liver disease worldwide with an estimated prevalence of 25–30% of the general population^3, 4^. All patients with NAFLD exhibit an increased risk of death linked to type 2 diabetes mellitus and cardiovascular risk factors^5^ and those with NASH have also an increased liver-related mortality due to the progression to cirrhosis and its complications including hepatocellular carcinoma^3^.

Currently available treatment options for NASH are limited to promotion of lifestyle changes in diet and exercise habits as well as to control of comorbidities such as diabetes mellitus and dyslipidemia^6, 7^. Among existing medications, only vitamin E and pioglitazone are recommended in selected patients although their long-term benefit has not been demonstrated and safety issues limit their use due to potentially unwanted side-effects^6^. Although a number of novel agents have recently entered clinical trials, availability of effective drugs to treat NASH is remains an unmet need^7, 8^.

In recent years, there has been a renewed interest in the therapeutic potential of herbal remedies for liver diseases including NAFLD^9^. Interestingly, pre-clinical studies with some of these agents have shown that they are able to modulate insulin resistance, lipid metabolism, oxidative stress, inflammation, and necro-apoptosis, all key factors in NAFLD/NASH pathogenesis^10^. However, translation of this knowledge into the clinic has been rather limited.

Among the herbal species tested experimentally in models of liver diseases *Andrographis paniculata* have been used in ancient oriental and Ayurvedic medicine to treat a myriad of disorders. The major bioactive phytoconstituent of *Andrographis paniculata* is Andrographolide (ANDRO), which is thought to be responsible for most of the biological effects of this herbal medicine^11^. ANDRO is a diverse molecule that possesses a broad range of biological activities, including anti-inflammatory, antitumor, antidiabetic, antibacterial, antimalarial, and hepatoprotective properties^12, 13^. ANDRO’s mechanism of action are also diverse and include antioxidant capacity, modulation of nitric oxide and prostaglandin production, modulatory action of immune cells and inhibitory effects on NF-kappa B (NF-*κ*B) transcription factor^12, 14^.

Several reports have shown hepatoprotective ANDRO effects in different experimental models such as carbon tetrachloride-induced hepatotoxicity, bile duct ligation and Concanavalin A and acetaminophen-induced liver injury (reviewed in ref. 15). With regard to NAFLD/NASH in particular, data on the potential beneficial effects of ANDRO is scarce. Of note, a recent report by Ding *et al*.^16^ showed that ANDRO was effective in preventing fat accumulation in liver of high-fat diet fed mice through regulation of sterol regulatory element-binding proteins (SREBPs) target genes. However, these authors did not explore further the effects of ANDRO on either inflammation or fibrosis in their animals due to shortcomings of the experimental models used.

Interestingly, due to its mechanisms of action in inflammatory disorders, ANDRO may be a suitable agent for reducing liver injury in the setting of NASH. In particular, ANDRO forms a covalent adduct with NF-κB, thus blocking the binding of NF-κB to nuclear proteins^14^. Since NFkB seems to be a critical player in NASH progression due to its central role in inflammation control by regulating the inflammatory cells infiltration as well as the expression of pro-inflammatory cytokines in both hepatocytes and Kupffer cells^17, 18^, targeting this pathway maybe of potential benefit in NASH. Of note, NFkB controls interleukin-1β (IL-1β) production, which is one of the most important cytokine involved in NASH progression^19^. Activation of IL-1β results from an enzymatic process driven by Caspase-1 (CASP-1), which in turn depends of a multi-protein complex, the inflammasome, which have recently been involved in NASH pathogenesis^20, 21^. Thus, due to its interaction with NF-κB, ANDRO should likely influence this inflammatory pathway in NASH. Moreover, it seems of interest to explore if ANDRO has any effects on the inflammasome pathway in NASH given recent evidence have linked ANDRO with Nucleotide-binding oligomerization domain leucine-rich-repeat containing receptor 3 (NLRP3) inflammasome inhibition in a mouse model of intestinal inflammation^22^. In the present study, we chose the choline-deficient amino acid-defined (CDAA) diet to mimic human NASH^23^ and studied the effects of ANDRO supplementation in this model. We found that ANDRO treatment ameliorates NASH development exercising and anti-inflammatory and anti-fibrotic action. In addition, we observed that ANDRO administration is associated with inflammasome inhibition, which is likely involved in the beneficial effects of this herbal compound in experimental NASH.

## Material and Methods

### Animals and diets

All experiments followed guidelines for the care and use of laboratory animals^24^ and all procedures were approved by the local institutional animal care and use committee (Comité de ética y bienestar Animal, Escuela de Medicina, Pontificia Universidad Católica de Chile [CEBA] resolution # 13-016). All efforts were made to minimize animals suffering and to reduce the number of animals used. Male C57bl6 mice were purchased from Jackson Laboratories (Bar Harbor, ME). Mice were aged 10 weeks at the beginning of this study and divided into four experimental groups (n = 7–10) receiving either CDAA diet (Catalog # 518753, Dyets Inc. Bethlehem, PA) to induce NASH^23^ or the choline-supplemented L-amino acid defined (CSAA, Catalog # 518754, Dyets Inc. Bethlehem, PA) diet as control and treated or not with ANDRO (Sigma-Aldrich, MO, Catalog # 365645). ANDRO was administered by intraperitoneal injection at a dose of 1 mg/kg three times per week as previously described^25^. Each group of animals were housed in transparent polycarbonate cages and subjected to 12 hours light/darkness cycles under a temperature of 21 °C and a relative humidity of 50%. Feeding and ANDRO administration lasted for 22 weeks. After ending the feeding course, mice were anesthetized (ketamine 60 mg/kg plus xylazine 10 mg/kg intraperitoneally) and then euthanized by exsanguination. Serum, liver and visceral adipose tissue samples were collected and processed or stored at −80 °C until analyzed.

### Histological and immunohistochemical studies

Liver histopathology was analyzed in paraformaldehyde-fixed liver sections which were stained with hematoxylin/eosin. Liver steatosis was specifically assessed by Oil-red-o staining (Abcam, USA) in frozen 7 μm liver criosections. Liver fibrosis was assessed using Sirius Red staining and red stained collagen fibers were quantitated by digital image analysis (ImageJ, NIH, US) as previously described^23^. Steatosis, inflammation, and ballooning were graded blindly by an experienced pathologist (J.T.) using the NAFLD activity score (NAS)^26^. Specifically, the amount of steatosis (percentage of hepatocytes containing fat droplets) was scored as 0 (<5%), 1 (5–33%), 2 (>33–66%) and 3 (>66%). Hepatocyte ballooning was classified as 0 (none), 1 (few) or 2 (many cells/prominent ballooning). Foci of lobular inflammation were scored as 0 (no foci), 1 (<2 foci per 200× field), 2 (2–4 foci per 200× field) and 3 (>4 foci per 200× field). The NAFLD activity score (NAS) was computed from the grade of steatosis, inflammation and ballooning. Immunohistochemistry staining for F4/80, a 160 kD glycoprotein expressed by murine macrophages, (F4/80, AbD Serotec, Hercules, CA, USA) was performed in formalin-fixed, paraffin-embedded liver sections according to the manufacturer’s instructions.

### RNA isolation and quantitative real-time polymerase chain reaction

Total RNA was prepared from frozen liver tissues using SV total RNA isolation kit (Promega, Madison, WI, USA). The quantity and purity of RNA was verified by measuring absorbance at 260 and 280 nm. Further, the integrity of RNA was confirmed by electrophoresis on formaldehyde- denaturing agarose gel. For RT-PCR assays, total RNA (5 μg) was reverse-transcribed using SuperscriptTM First-Strand Synthesis System (Invitrogen, California, USA)^27^. The sequences of the primers used for quantitative PCR are given in Supplementary Table 1.

### Immunoblot analysis

Immunoblot analyses were performed as previously described^25^. Anti-Col1A, anti-CTGF, anti-Collagen type IV, anti- Apoptosis-Associated Speck-Like Protein Containing CARD (ASC), anti-CASP1, anti-CASP1p10, anti-NLRP3, anti-actin antibodies (Abcam, USA) were used in combination with appropriate peroxidase-conjugated secondary antibodies. Tubulin or Actin were used as a loading control (Sigma, USA). Bands were visualized with the enhanced chemiluminescence reagent (Thermo, USA) and digitized using a CCD camera (UVP, USA). Densitometric analysis and quantification were performed using ImageJ software (NIH, USA).

### Liver function tests

Serum alanine aminotransferase (ALT) was measured using a Merck Diagnostica Kit (Darmstadt, Germany). Hepatic triglycerides content (HTC) was measured using a kit from Human Gesselheit (Wiesbaden, Germany).

### Cell cultures and NFkB reporter assay

HepG2 cells (ATCC, USA) were routinely cultured with Dulbecco’s Modified Eagle’s Medium containing 10% fetal bovine serum. HepG2 cells were seeded into 12-well plates 24 h prior to treatments at approximately 60% confluence. To induce lipid overload HepG2 cells were exposed to palmitic Acid as previously described^28, 29^. Then, cells were stimulated with lipopolysaccharide (1000 ng/ml) for 24 h with or without the pre-incubation of 50 μM ANDRO for another 24 h bovine serum albumin and dimethyl sulfoxide were used as controls respectively. Cell viability after treatments was monitored by MTS Cell Proliferation Assay Kit (Abcam, USA). No change in viability was observed with the concentrations used in this study. To evaluate NF-κB activation, HepG2 cells were transfected with an NF-κB reporter gene using the Cignal NF-κB Reporter luciferase Kit (SaBiosciences-Qiagen). This kit consists in a mixture of inducible NF-κB -responsive firefly luciferase construct and constitutively expressing renilla luciferase construct, as well as positive and negative controls. The NF-κB -responsive luciferase construct encodes the firefly luciferase reporter gene under the control of a minimal CMV promoter and tandem repeats of the NF-κB transcriptional response elements. Fat-laden hepatocytes were transfected with the constructs using Lipofectamine 2000 (Invitrogen, USA) according to the manufacturer instructions. Each transfection contained 200 ng of NF-κB reporter or positive or negative control. The inducible pathway reporter and noninducible negative control are transfected and subjected to experimental treatments in parallel. After 24 hours, fat-laden transfected hepatocytes were treated as described above. The activity of firefly and renilla luciferases was determined using the Dual-Luciferase Reporter Assay System (Promega, USA) as described by the manufacturer. Dual-luciferase results were determined for each transfectant. The change in the activity was determined by comparing the normalized luciferase activities of the reporter in treated versus untreated transfectants.

### Cell Viability assay

Cell proliferation assays were conducted using the MTS assay kits (Abcam, USA) as described by the manufacturer. Cells, were treated as described above and then the media was replaced with a 10% v/v solution of MTS reagent in PBS. The plate was then incubated for 3 hours before obtaining readings.

### CASP-1 activation

Cells lysates were assayed for their ability to cleave a fluorescent CASP-1 substrate, following the manufacturer’s instructions (Abcam, USA).

### Isolation of hepatocytes, Kupffer cells, and hepatic stellate cells

In additional experiments we sought to assess the anti-inflammatory and antifibrotic effects of ANDRO *in vitro* using different liver cell populations. To that end, hepatocytes, Kupffer cells (KC), and hepatic stellate cells (HSC) were simultaneously obtained from pathogen-free male Wistar using *in situ* perfusion followed by density gradient cell separation as previously described^23, 30^. The effect of ANDRO on Cytokine-induced inducible nitric oxide synthase (iNOS*)* expression was assessed in hepatocytes by incubation of cells with a cytokine mixture (TNF-α, Interleukin-1 and interferon-γ [IFN-γ]) with and without ANDRO. In separate experiments, we also assessed ANDRO effects on lipopolysaccharide (LPS)-induced iNOS expression in hepatocytes. Similarly, the effect of ANDRO on LPS-induced TNF-α expression in Kupffer cells (LPS [0.1 ng/mL] was explored). Finally, to evaluate the potential modulatory effects of ANDRO on liver fibrogenesis, we assessed the effect of ANDRO (20 and 50 µM) on mRNA levels of α-smooth muscle actin (α-SMA), transforming growth factor beta (TGF-β) and collagen-type 1-α1 (Col1a1) in cultured HSC.

### Co-immunoprecipitation assay

Cells were lysed in RIPA buffer on ice (20 mM Tris · HCl, pH 7.4, 150 mM NaCl, 0.5% Nonidet P-40, 1% Triton X-100) containing 10 μg/ml aprotinin, 5 μg/ml leupeptin, 10 μg/ml phenylmethylsulfonyl fluoride, 1 μg/ml pepstatin, 2 mM EDTA, 2 mM EGTA, 2 mM sodium orthovanadate, 30 mM sodium pyrophosphate, and 100 mM sodium fluoride. Then were centrifuged at 12,000 g at 4 C for 15 min. Equal amounts of protein (300 μg) from precleared extracts were immunoprecipitated for 2 h at 4 °C with a 1 μg of anti-ASC antibody at 4 C overnight and precipitated with protein A/G-agarose beads (Santa Cruz, sc-2003) for another 4 h at 4 C. The beads were washed with the lysis buffer 4 times by centrifugation at 1,000 g at 4 C. Equal volumes of immunoprecipitated protein were separated by SDS-PAGE and western blot was performed with the indicated antibodies. Bound antibodies were visualized with horseradish peroxidase-coupled secondary antibodies (Thermo, USA) followed by development with an enhanced chemiluminescence system (Thermo, USA).

### Statistical analyses

Analyses were performed using GraphPad software (version 5.03, GraphPad Software Inc., CA, USA). The significance level was set at α = 5% for all comparisons. The statistical significance of differences between the means of the experimental groups was evaluated using one-way analysis of variance (ANOVA) with a post-hoc Bonferroni multiple-comparison test or two tailed t-tests. Data are expressed as mean ± SEM or as absolute number or percentage for categorical variables.

## Results

### ANDRO-treated mice are protected from CDAA-induced hepatomegaly and liver injury

**Figure 1:**
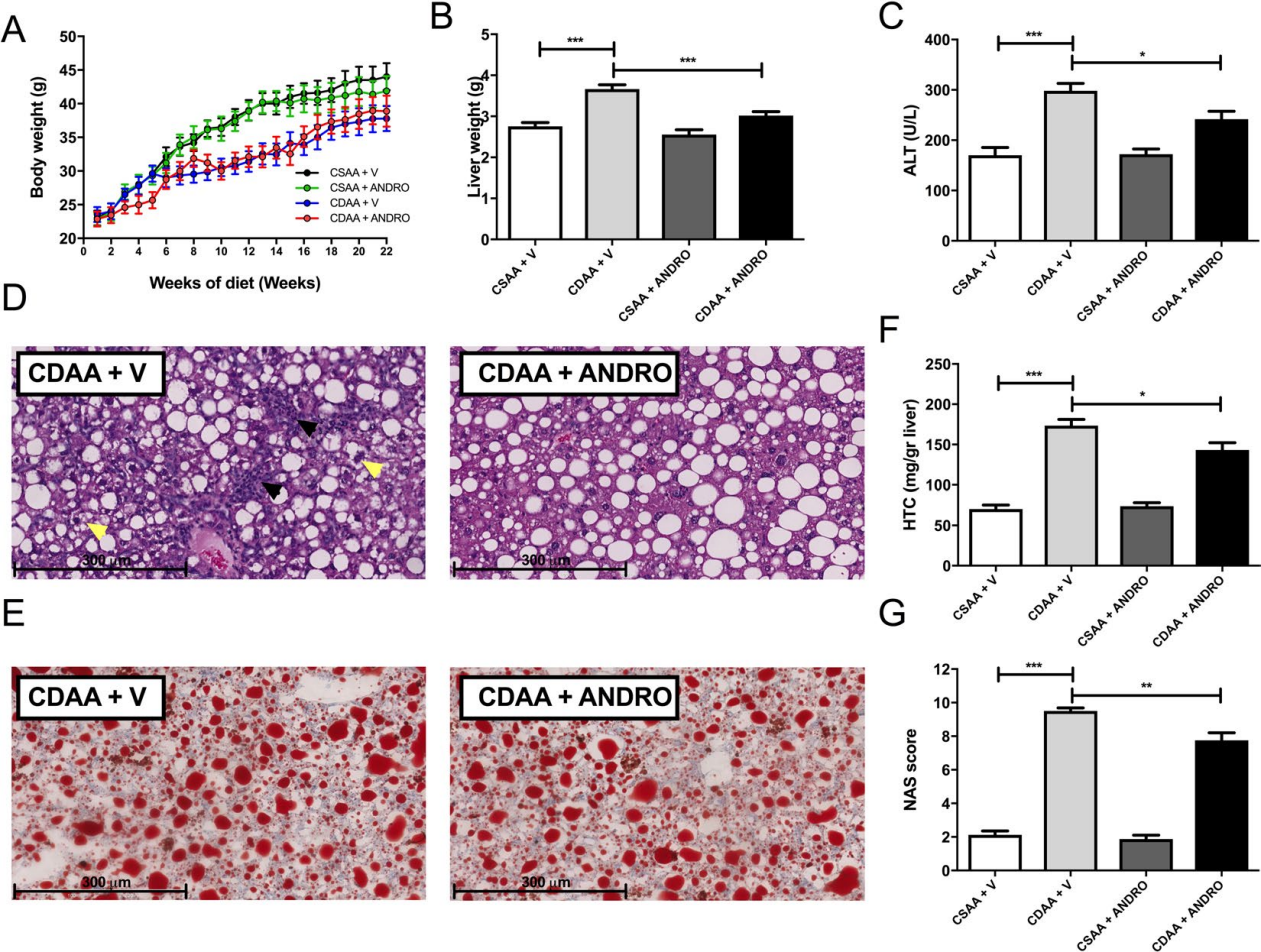
Andrographolide-treated mice are protected from CDAA-induced hepatomegaly and liver injury. Mice weaned after 21 days were placed on normal chow diet for 4 weeks and thereafter placed either on choline-deficient amino acid-defined (CDAA) or choline-supplemented L-amino acid defined (CSAA) diets for an additional 22 weeks. During this period mice were injected with AP three times per week (1 mg/kg, intraperitoneally). (**A**) Body weight gain, (**B**) Liver weight, (**C**) alanine aminotransferase (ALT) serum levels, (**D**) Histological evaluation of liver tissue (hematoxylin/eosin staining), inflammatory foci and hepatocyte ballooning are indicated by black and yellow arrows respectively. (**E**) Hepatic triglyceride content (HTC). (**F**) Oil-red-O staining of liver samples. (**G**) Non-alcoholic activity score (NAS) as assessed by a pathologist in a blindly fashion. Data are shown as means ± SEM. Statistical significance was evaluated using ANOVA with a post-hoc Bonferroni multiple-comparison test (*p ≤ 0.05, **p ≤ 0.01 and ***p ≤ 0.001).

ANDRO and vehicle-treated mice fed a CDAA diet or control diet for 22 weeks showed significant weight gain throughout the experimental period (Fig. 1A). Of note, there were no significant differences in body weight after 22 weeks of feeding among different experimental groups. On the other hand, CDAA diet was associated with a significant increase in liver weight, which was prevented by ANDRO treatment (Fig. 1B). In addition, serum ALT levels were significantly elevated in vehicle-treated mice fed with CDAA in comparison to vehicle-treated mice on CSAA diet (p ≤ 0.001). Also, ANDRO-treated mice showed lower levels of serum ALT compared with vehicle treated mice (Fig. 1C) (p ≤ 0.05).

Liver histological examination (hematoxylin & Eosin staining) showed less inflammatory foci (Fig. 1D, black arrows) and hepatocyte ballooning (Fig. 1D, yellow arrows) in CDAA diet-fed mice treated with ANDRO compared to controls. Although liver steatosis was present in all groups and appear to be of similar grade in CDAA diet-fed mice with and without ANDRO treatment in histological slides stained with Oil-red-O (Fig. 1E), HTC was significantly lower in ANDRO-treated mice fed with CDAA diet compared with vehicle-treated animals fed with the same diet (Fig. 1F) (p ≤ 0.01). In addition, evaluation of liver histological samples using the NAS system^31^, showed lower scores in ANDRO-treated mice fed with the CDAA diet (Fig. 1G) (p ≤ 0.01).

### ANDRO treatment attenuates liver fibrosis development in CDAA-induced NASH

**Figure 2:**
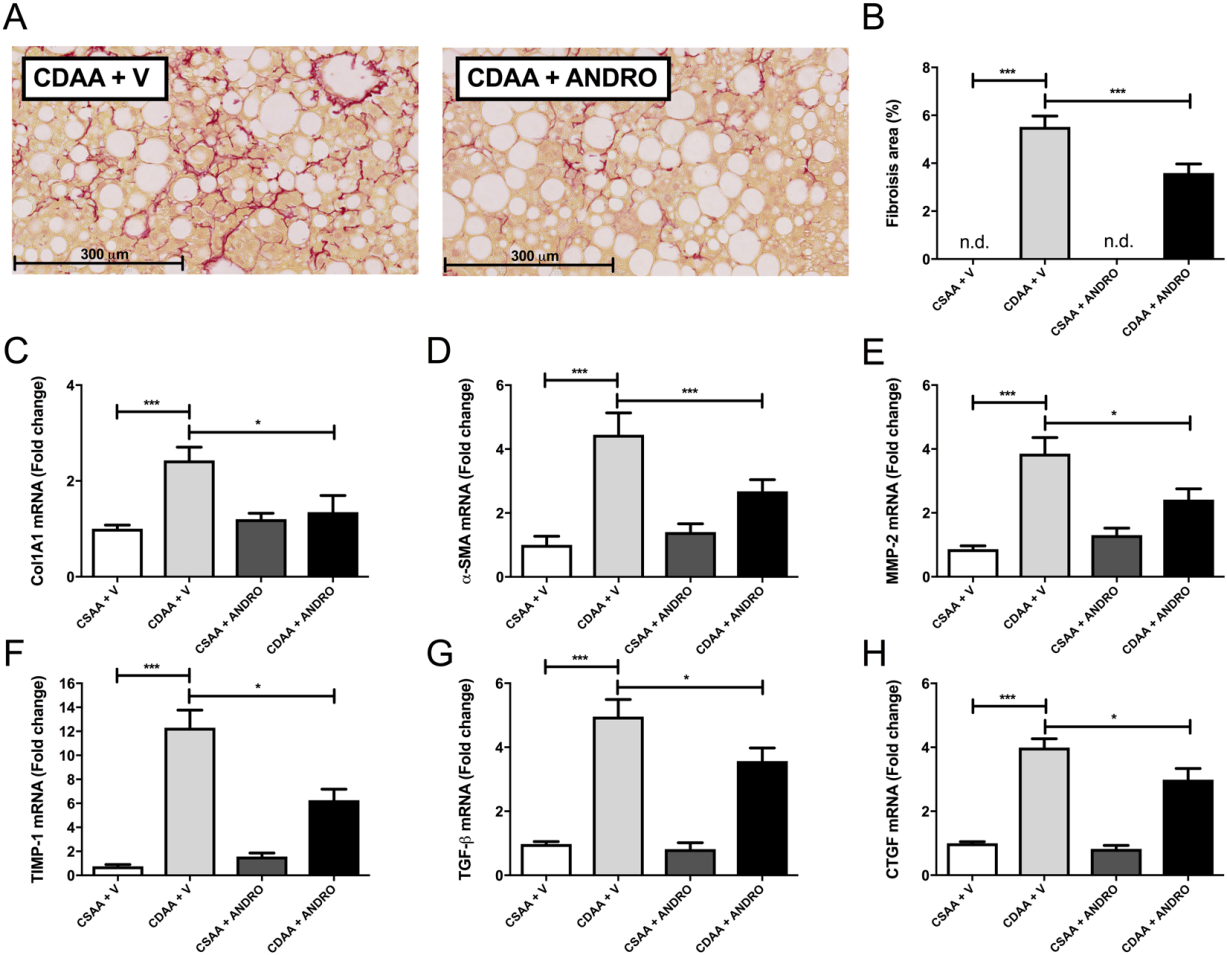
Andrographolide(ANDRO)-treated mice are protected from CDAA-induced liver fibrosis. (**A**) Liver sections Sirius Red staining from mice fed with choline-deficient amino acid-defined (CDAA) or choline-supplemented L-amino acid defined (CSAA) diets during 22 weeks and treated with ANDRO or Vehicle (V) as described in material and methods. (**B**) Fibrosis area was quantified using digital image analysis of the red-stained area in Sirius red-stained samples (NIH, Bethesda, MD, USA) and mRNA levels of Collagen, type I, alpha 1 (**C**), Alpha-smooth muscle actin (α-SMA) (**D**), matrix metalloproteinase-2 (MMP-2) (**E**), Tissue inhibitor of metalloproteinase-1 (TIMP-1) (**F**), transforming growth factor beta TGF-β, (**G**) and Connective tissue growth factor precursor (CTGF) (**H**) assessed by real time-PCR. Statistical significance was evaluated using ANOVA with a post-hoc Bonferroni multiple-comparison test (*p ≤ 0.05 and ***p ≤ 0.001. n.d = not detected).

**Figure 3:**
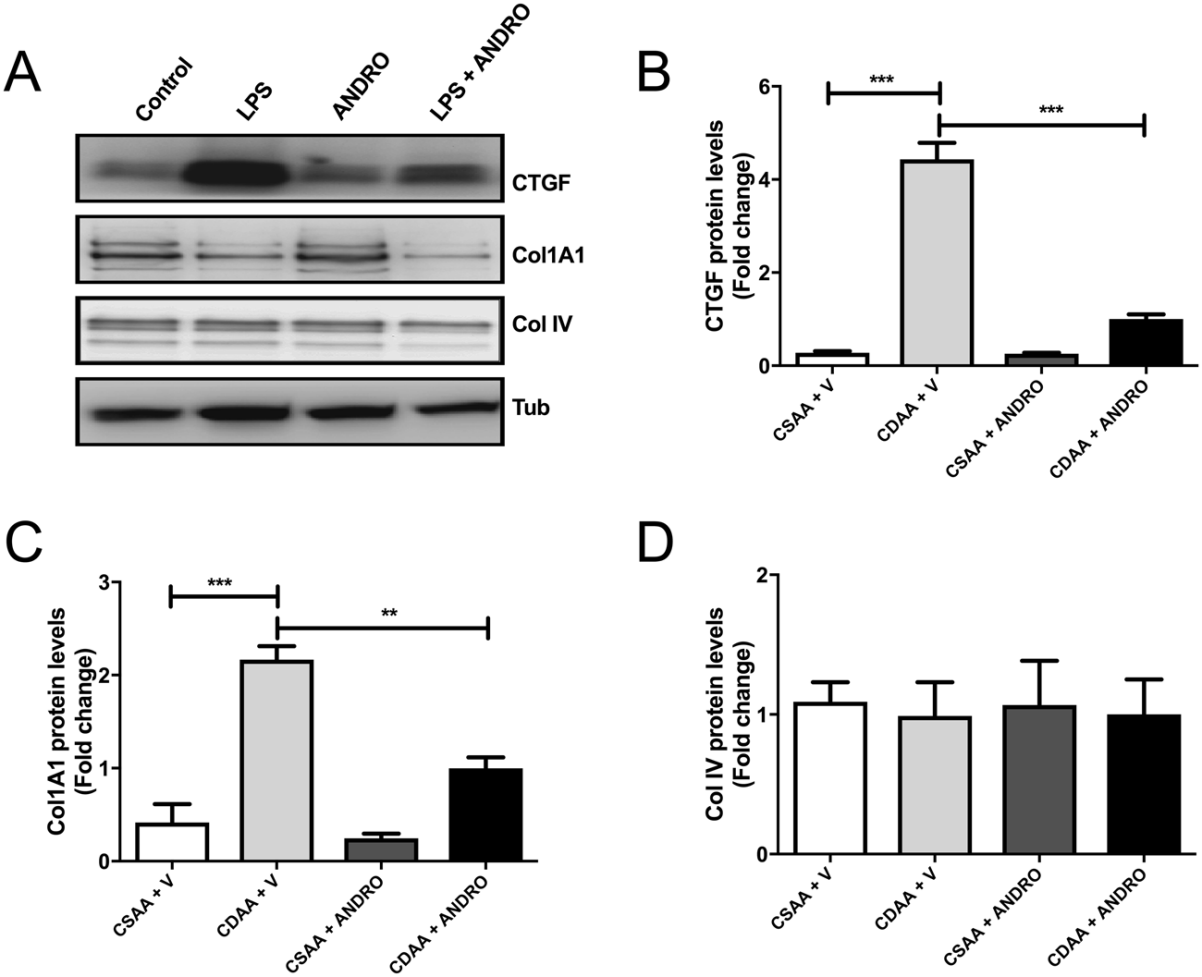
Effect of Andrographolide (ANDRO) on hepatic fibrogenic proteins. Total proteins were isolated from livers of mice fed either a choline-deficient amino acid-defined (CDAA) or choline-supplemented L-amino acid defined (CSAA) diets treated or not with ANDRO, as described in material and methods. (**A**) Representative Western blot analysis of connective tissue growth factor (CTGF), collagen type I, alpha 1 chain (COL1A1) and collagen IV (Col IV). Tubulin (Tub) was used as loading control. Densitometric analysis of CTGF (**B**), COL1A1 (**C**) and Collagen IV (**D**) is shown in the graphs (n = 3–4 animals per group). Statistical significance was evaluated using ANOVA with a post-hoc Bonferroni multiple-comparison test (**p ≤ 0.01 and ***p ≤ 0.001).

As mentioned previously, CDAA diet feeding for 22-weeks induced a full-blown histological picture of NASH in the mouse liver with significant fibrosis as shown by conventional Sirius Red staining and digital image analysis (Fig. 2A,B). Notably, ANDRO-treated mice exhibited a reduced collagen deposition area compared with controls indicating an anti-fibrotic action of the compound (Fig. 2A,B) (p ≤ 0.001). Also, as shown in Fig. 2C–H, ANDRO administration significantly attenuates the increase in hepatic mRNA levels of several markers of fibrogenesis induced by the CDAA diet including Collagen type I, alpha 1 (COL1A1, p ≤ 0.001), Alpha-smooth muscle actin (α-SMA, p ≤ 0.001) matrix metalloproteinase-2 (MMP-2), Tissue inhibitor of metalloproteinase-1 (TIMP-1), transforming growth factor beta TGF-β and Connective tissue growth factor (CTGF, p ≤ 0.001). Confirmatory experiments assessing protein levels of CTGF and Col1A1 showed that, in agreement with mRNA data, ANDRO treatment was associated to reduced protein mass of both fibrogenic markers (Fig. 3A–C). Interestingly, levels of collagen IV, a non-fibrotic extracellular matrix protein, remained unchanged in ANDRO-treated animals (Fig. 3D). Collectively, these results show that ANDRO-treatment exerts an antifibrotic action in experimental NASH.

### ANDRO reduces macrophage infiltration in CDAA-fed mice

**Figure 4:**
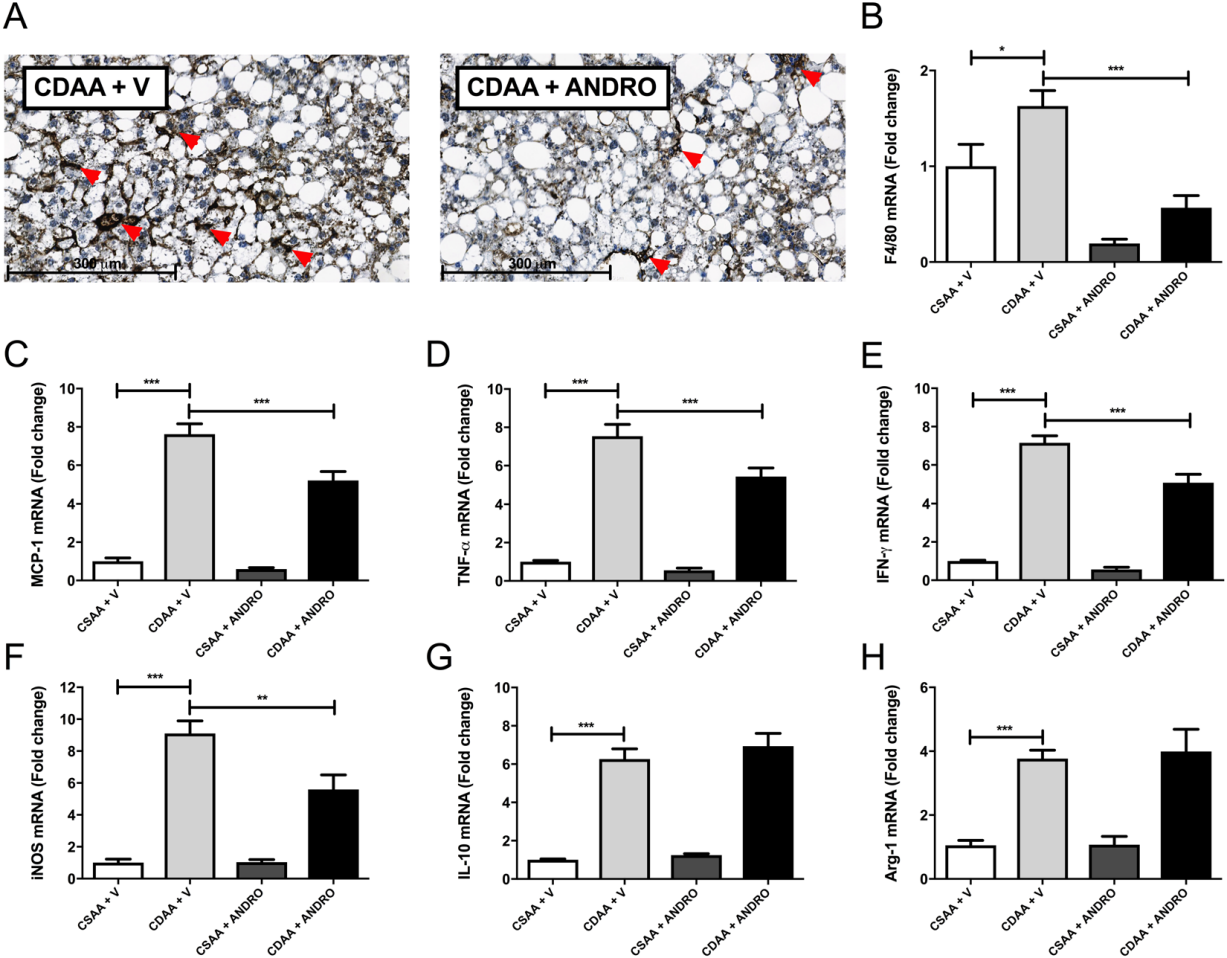
Andrographolide (ANDRO) alleviates CDAA-diet-induced inflammation. Mice were fed with choline-deficient amino acid-defined (CDAA) or choline-supplemented L-amino acid defined (CSAA) diets during 22 weeks and treated with ANDRO or Vehicle (V) as described in material and methods. (**A**) Immunohistochemistry of liver sections using anti-F4/80 antibody, red arrows show inflammatory foci. Hepatic mRNA levels of **(B)** F4/80, **(C)** Monocyte chemotactic protein 1 (MCP-1), **(D)** Tumor necrosis factor-alpha (TNF-α), **(E)** Interferon gamma (IFNγ), **(F)** inducible nitric oxide synthase (iNOS), **(G)** interleukin-10 (IL-10) and **(H)** arginase-1 (Arg-1) were also assessed by real time-PCR. Statistical significance was evaluated using ANOVA with a post-hoc Bonferroni multiple-comparison test (*p ≤ 0.05, **p ≤ 0.01 and ***p ≤ 0.001).

To better characterize the anti-inflammatory action of ANDRO in NASH, we assessed macrophage infiltration by immunohistochemistry detecting F4/80 positive cells in liver tissue collected from CDAA fed mice with or without simultaneous ANDRO administration. Figure 4A shows that CDAA diet fed mice treated with ANDRO exhibit less macrophage hepatic infiltration as assessed by immunostaining (red arrows), which correlated with a reduced F4/80 mRNA expression (Fig. 4B) (p ≤ 0.001). In addition, mRNA levels of the monocyte chemoattractant protein-1 (MCP-1), a marker of macrophage activation^32^, were decreased in CDAA diet fed mice treated with ANDRO (Fig. 4C) (p ≤ 0.001). Also, the induction of the expression of M1 inflammatory cytokines [Tumor necrosis factor-alpha (TNF-α), Interferon gamma (IFNγ) and iNOS], by CDAA diet was prevented by ANDRO-treatment (Fig. 4D–F) (p ≤ 0.001). Of note, the expression of interleukin-10 (IL-10) and arginase-1 (Arg-1) were not affected (Fig. 4G,H) (p > 0.05), suggesting a specific effect of ANDRO on M1 phenotype of macrophage polarization, which is regarded as essentially proinflammatory^32^.

### ANDRO treatment reduces inflammasome activation by CDAA diet

**Figure 5:**
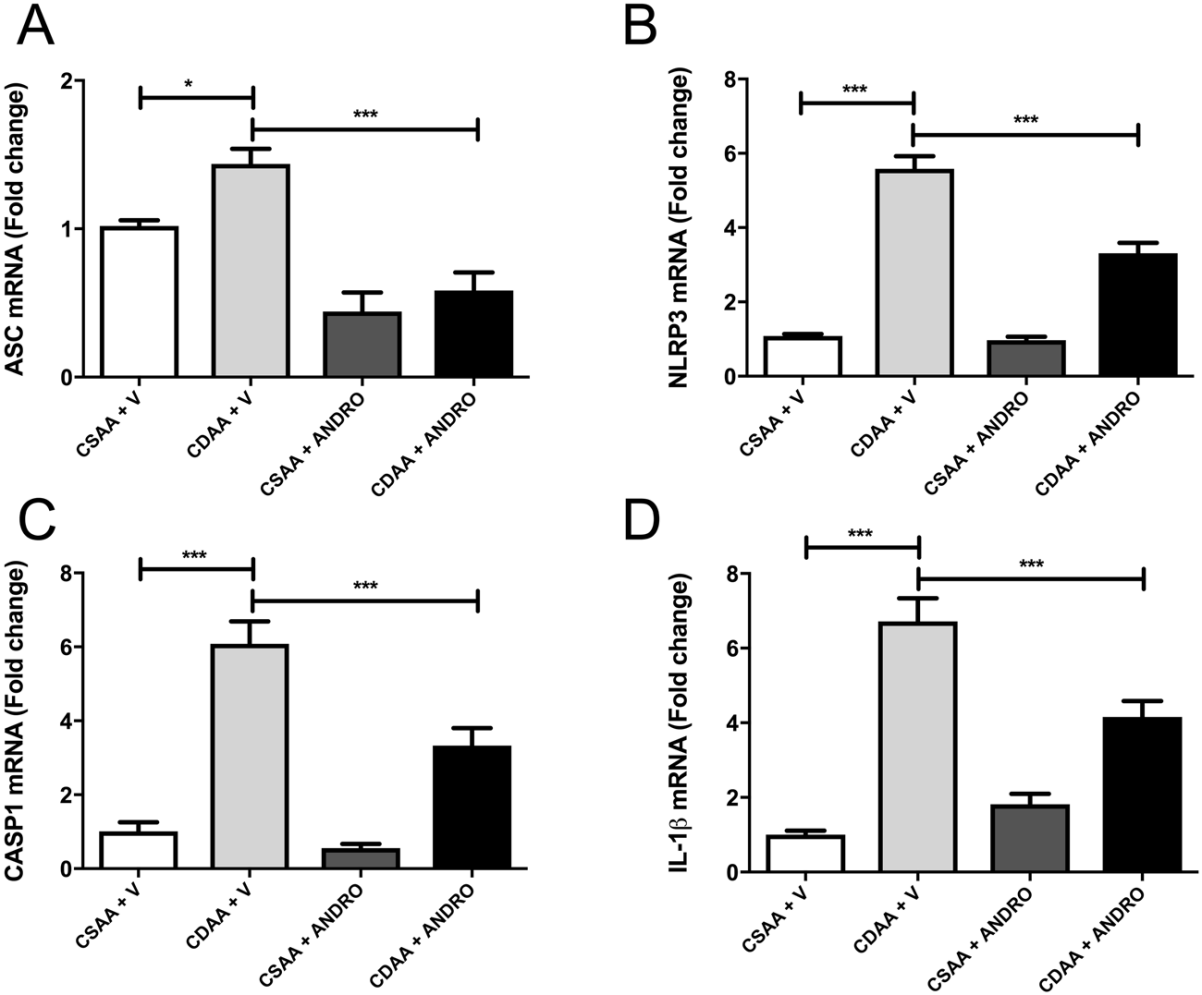
Andrographolide (ANDRO) modulates CDAA-diet-induced inflammasome activation. Mice were fed with choline-deficient amino acid-defined (CDAA) or choline-supplemented L-amino acid defined (CSAA) diets during 22 weeks and treated with ANDRO or Vehicle (V) as described in material and methods. RNA was isolated from liver tissue and mRNA levels of (**A**) anti-Apoptosis-Associated Speck-Like Protein Containing CARD (ASC), **(B)** Nucleotide-binding oligomerization domain leucine-rich-repeat containing receptor 3 (NLRP3), **(C)** Caspase-1 (CASP1) and **(D)** interleukin-1β (IL-1β) were assessed by real time PCR. Statistical significance was evaluated using ANOVA with a post-hoc Bonferroni multiple-comparison test (*p ≤ 0.05 and ***p ≤ 0.001).

Assessment of the expression of main inflammasome components (ASC [Apoptosis-associated speck-like protein] and NLRP3) in experimental groups showed that mRNA expression of both inflammasome components (ASC and NLRP3) was significantly reduced by ANDRO treatment (Fig. 5A,B) (p ≤ 0.001). ANDRO treatment, also reduced IL-1β and caspase-1 expression (Fig. 5C,D) (p ≤ 0.001), suggesting that ANDRO can modulate NASH progression by regulating the inflammasome.

### NFkB inhibition by ANDRO treatment is correlated with inflammasome inhibition

**Figure 6:**
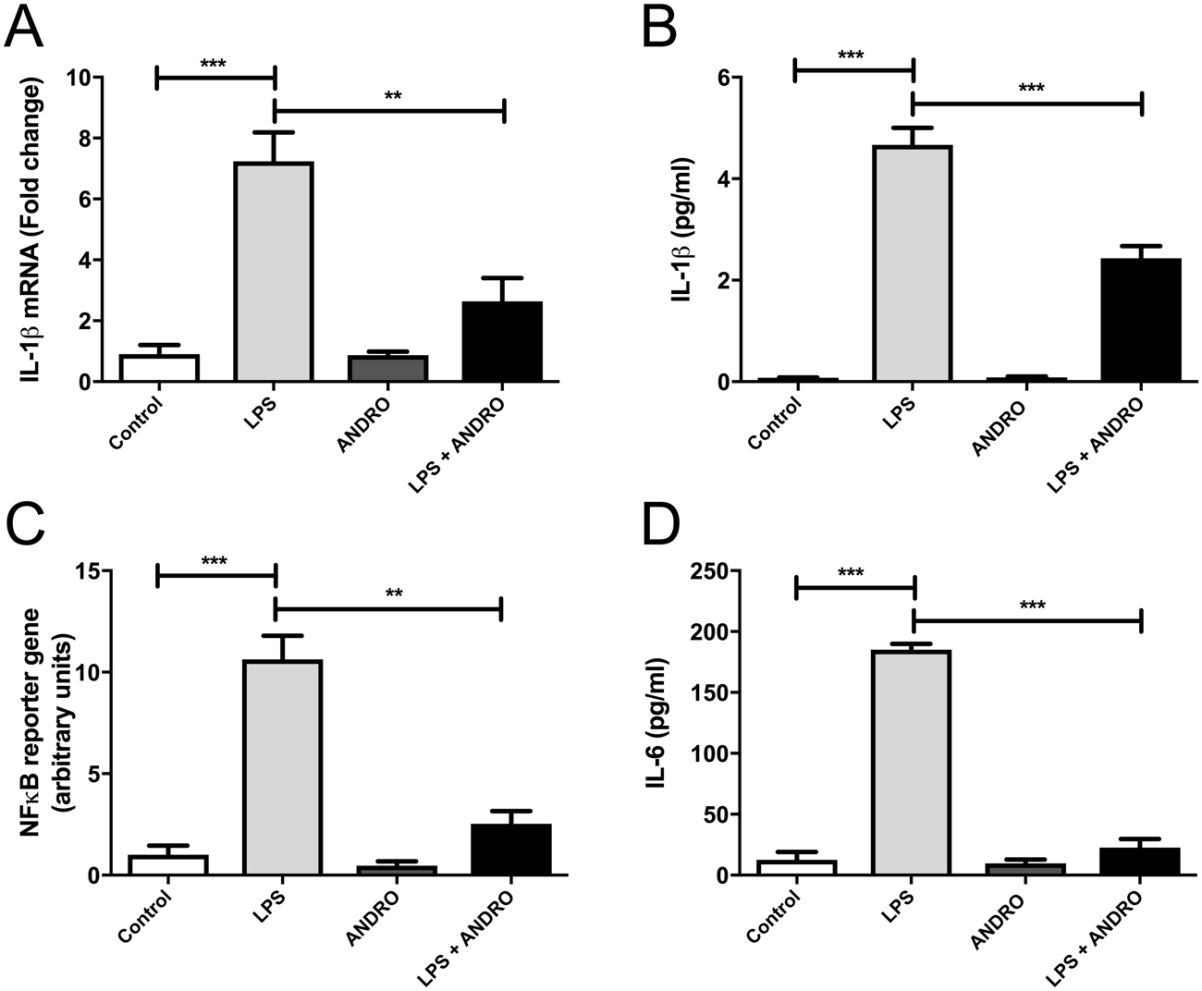
Andrographolide (ANDRO) inhibits IL-1β expression through NFκB inhibition. HepG2 cells were incubated for 24 h with palmitic acid to induce lipid droplet accumulation. Then, cells were stimulated with Lipopolysaccharides (LPS) (1000 ng/ml) for 24 h in the presence or absence of ANDRO (50 μM). **(A)** interleukin-1β (IL-1β) gene expression. **(B)** IL-1β protein levels in the supernatant fraction were analyzed by ELISA. **(C)** Fat-laden HepG2 cells were co-transfected with NFκB transcription factor-responsive firefly luciferase and Renilla luciferase constructs and treated with LPS (1000 ng/ml), ANDRO (50 μM) or both. **(D)** IL-6 protein levels in the supernatant fraction. Statistical significance was evaluated using ANOVA with a post-hoc Bonferroni multiple-comparison test (**p ≤ 0.01 and ***p ≤ 0.001).

**Figure 7:**
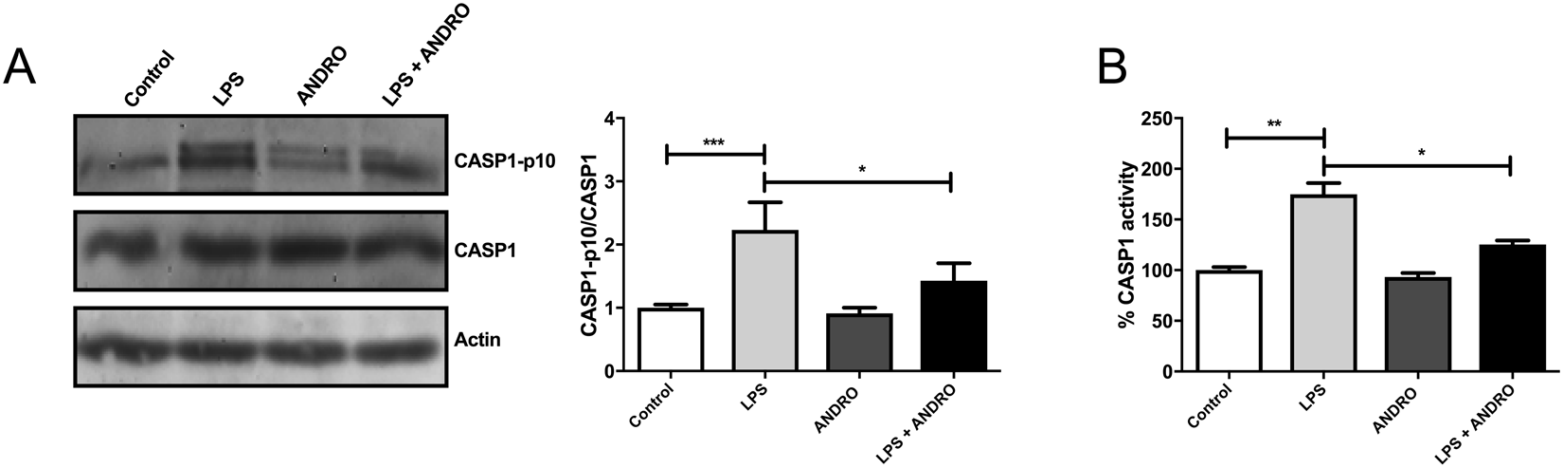
Andrographolide (ANDRO) inhibits Caspase-1 (CASP1) activation and interleukin 1β (IL1 β) maturation *in vitro*. Primary hepatocytes isolated from C57bl6 mice were incubated for 24 h with palmitic acid to induce lipid droplet accumulation. Then, cells were stimulated with Lipopolysaccharides (LPS) (1000 ng/ml) for 24 h in the presence or absence of ANDRO (50 μM). **(A)** Protein levels of CASP1 and cleaved CASP1 (CASP1-p10) were determined by Western blot as described in material and methods. Actin was used as loading control. The CASP1-P10/CASP1 ratio was calculated to assess the generation of active CASP1. **(B)** CASP-1 activity as evaluated by an enzymatic assay. Statistical significance was evaluated using ANOVA with a post-hoc Bonferroni multiple-comparison test (*p ≤ 0.05, **p ≤ 0.01 and ***p ≤ 0.001).

To evaluate if ANDRO have a direct effect on inflammasome activation in fat-laden liver cells we explored if ANDRO treatment influenced IL-1β expression *in vitro*. To that end, HepG2 cells were treated with palmitic acid to induce fat overload as described^29^. Then, we stimulate the cells with LPS to activate the inflammasome in the presence or absence of ANDRO. Figure 6A,B shows that ANDRO inhibits IL-1β expression and secretion induction by LPS in fat-laden HepG2 cells respectively (p ≤ 0.001). In the same model, ANDRO determined a significant reduction of NF-κB activity (Fig. 6C, p ≤ 0.001) suggesting that ANDRO could regulate the inflammasome through NF-κB inhibition. Of note, IL-6, a NF-κB–dependent cytokine, was also reduced by ANDRO (Fig. 6D, p ≤ 0.001) confirming ANDRO NF-κB-inhibiting activity^12^. To evaluate, if ANDRO had a direct effect on the inflammasome, we examined the NLRP3 inflammasome activation in fat-laden hepatocytes after 24 h of LPS incubation. As shown in Fig. 7A, activation of CASP1 by LPS (as indicated by the presence of the cleaved form CASP1-p10) was significantly inhibited by ANDRO. Besides, ANDRO markedly inhibited CASP-1 enzymatic activity (Fig. 7B).

**Figure 8:**
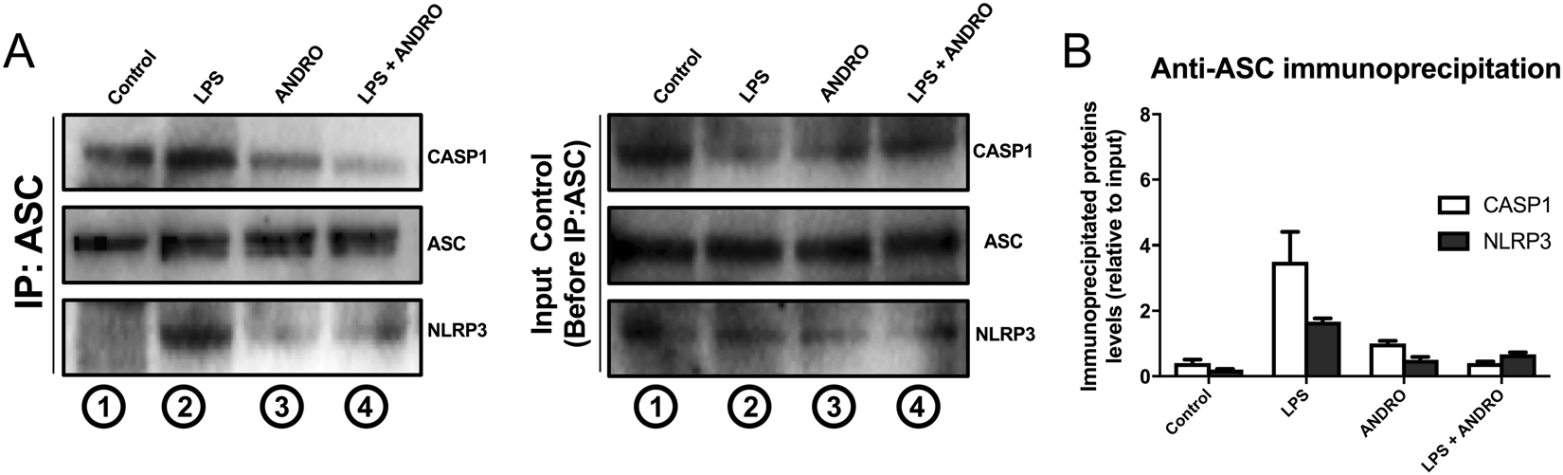
Andrographolide (ANDRO) affects inflammasome assembly in hepatic cells. Fat-laden hepatocytes were treated with LPS for a short period of time (3 h) and then were treated with ANDRO for 1 h. Total proteins were isolated and immunoprecipitated with an antibody against the inflammasome protein Apoptosis-Associated Speck-Like Protein Containing CARD (ASC). (**A**) The immunoprecipitated extract was separated by SDS-PAGE and then blotted and revealed with antibodies anti-Nucleotide-binding oligomerization domain leucine-rich-repeat containing receptor 3 (NLRP3) and Caspase-1 (CASP1) to assess inflammasome assembly (IP:ASC, left panel). CASP1 and NLRP3 immunoprecipitated with ASC in LPS-treated cells (IP:ASC, left panel, lane2) confirming LPS-induced inflammasome assembly. ANDRO treatment interfered with this process (IP:ASC, left panel, lane4). Before the immunoprecipitation assay, the presence of the indicated proteins was determined by western blot of total extracted proteins (Input control blot, right panel). **(B)** Anti-ASC immunoprecipitated proteins (CASP1 and NLRP3) were quantified and plotted over input protein levels.

Furthermore, we addressed the question if ANDRO affects inflammasome assembly. To that end, we performed immunoprecipitation analysis after short incubations with LPS (3 h) to avoid significant variations in the inflammasome protein levels (Fig. 8A). Also, we evaluated total basal protein levels by western blot previous to start the immunoprecipitation assay (Fig. 8B, Input blot). Immunoprecipitation experiments confirmed that LPS promoted inflammasome assembly since LPS treatment determined co-precipitation of CASP-1 and NLRP3 with ASC (Fig. 8A, lane 2) and that ANDRO influenced this process (Fig. 8A, lane 4). Basal levels of ASC and NLRP3 remained unchanged, but CASP1 seems to be decreased, probably due to CASP1 cleavage induced by LPS. (Fig. 8B).

### Effects of ANDRO hepatocytes, KC, and HSC

**Figure 9:**
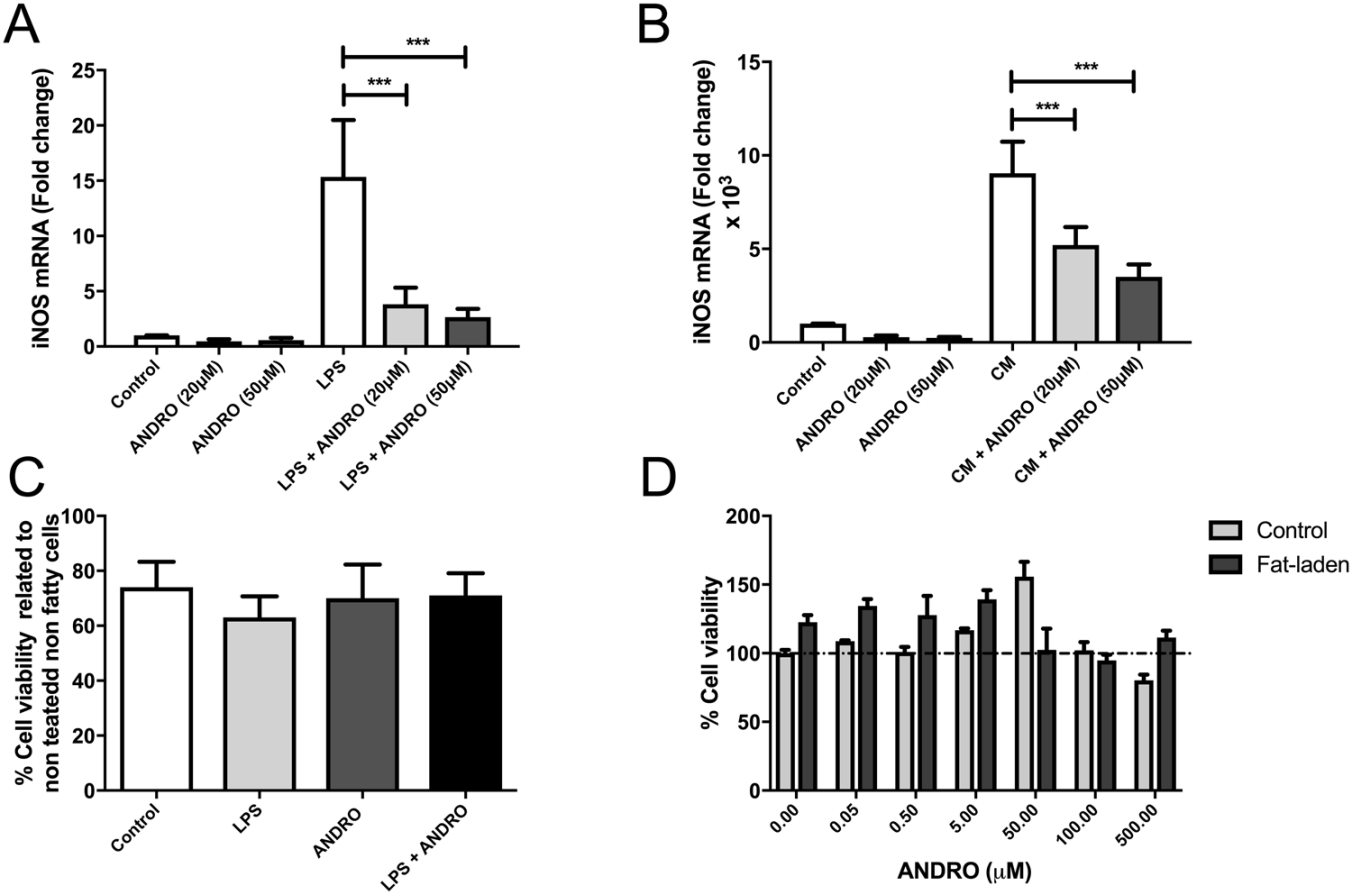
Andrographolide (ANDRO) inhibits inducible nitric oxide synthase (iNOS) expression in hepatocytes. iNOS gene expression was evaluated in freshly isolated hepatocytes that were incubated for 24 hours with LPS **(A)** or a Cytokine Mixture **(B)** containing TNF-α, Interleukin-1 and interferon-γ in the presence or absence of different concentrations of ANDRO. To evaluate cell viability under the experimental condition a MTS cell proliferation assay **(C)** and a dose-response curve **(D)** were performed in these settings. The statistical significance was evaluated using ANOVA with a post-hoc Bonferroni multiple-comparison test (***p ≤ 0.001).

**Figure 10:**
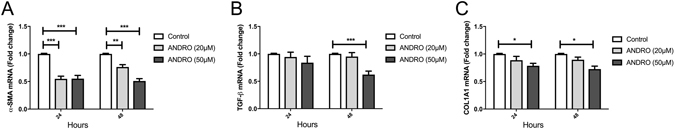
Andrographolide (ANDRO) modulates expression of fibrogenic genes in HSC. HSCs were isolated from male Wistar rats as described in material and methods section. Cells were incubated at different time points (24 and 48 hours) with two different AP concentrations (20 and 50 μM). Then, mRNA expression of relevant hepatic fibrogenic genes [Alpha-smooth muscle actin (α-SMA), transforming growth factor beta (TGF-β) and collagen type I, alpha 1 chain (COL1A1)] was assessed. The statistical significance was evaluated using ANOVA with a post-hoc Bonferroni multiple-comparison test (*p ≤ 0.05, **p ≤ 0.01, ***p ≤ 0.001).

As shown in Fig. 9A,B, ANDRO significantly reduced both Cytokine-induced and LPS-induced iNOS expression in primary hepatocytes. In separate experiments we confirmed that the concentrations of ANDRO used in this study did not affect cell viability (Fig. 9Cy,D). The same was observed for LPS-induced TNF-α expression in KC (data not shown). Finally, in experiments involving HSC, ANDRO significantly reduced the expression of α-SMA (Fig. 10A) at both concentrations studied and the expression of TGF-β and COL1A1 in HSC when used at 50 μM (Fig. 10B,C).

## Discussion

NASH can lead to advanced liver fibrosis and cirrhosis, which eventually results in liver failure^3^. To date, effective pharmacologic agents and therapeutic approaches for this condition are limited^6, 33^. In this context, the use of herbal compounds for the treatment of this disease has expanded in recent years^34^. However, scientific information on the efficacy and mechanisms of action of these natural compounds is scarce, which limits further therapeutic development of novel agents. In this study, we have demonstrated that ANDRO, a botanically-derived compound, exerts beneficial effects in a mouse model of NASH including attenuation of inflammation and fibrosis.

The anti-inflammatory and anti-fibrotic effects of ANDRO in experimental NASH could be attributed to ANDRO modulatory effects of multiple signaling pathways in the liver. Among them ANDRO modulatory effects of NF-κB-related pathways^13^ may play a role since ANDRO suppressed NF-κB transcriptional activation in HepG2 cells transfected with a NF-κB2 luciferase reporter (Fig. 6). NF-κB plays a key role in liver inflammation and hepatic immune response due to its influence in in different hepatic cell populations^17^ and the inhibition of NF-κB activation by ANDRO, through covalent modification of the p50 subunit of this transcription factor, has been described as a unique pharmacological mechanism explaining ANDRO’s anti-inflammatory actions^14^. In the present study, we clearly documented ANDRO modulation of inflammatory and fibrotic responses both *in vivo* and *in vitro* and showed that ANDRO markedly influences NF-κB and inflammasome pathways in hepatocyte-derived cell lines thus supporting that ANDRO acts, at least in part, through its effects in these latter pathways. Also, given the attenuation of inflammatory responses in KC as well as modulation of fibrogenesis in HSCs and that has been shown that NF-κB critically influences the inflammatory response of KC, and survival and activation in HSCs^17, 18^, it is likely that ANDRO exerts its action through this pathway. While these findings are correlative, they provide the foundational basis for future studies attempting to dissect specifically ANDRO’s mechanism of action in NAFLD/NASH.

In the present study we also explored if ANDRO influences the expression of inflammasome components in experimental NASH. The inflammasome consists of an intracellular multi-protein complex that is formed upon exposure of a wide range of stimuli generated during liver injury and sterile inflammation^35^. The nucleotide oligomerization domain (NOD)-like receptors (NLRs), that belong to the pattern recognition receptors family, recognize damage associated molecular patterns (DAMPs) and pathogen associated molecular patterns (PAMPs) signals and promotes the assembly of inflammasome proteins^21^. In turn, the inflammasome activates CASP1 that cleaves pro-IL-1β into the mature form of IL-1β determining amplification of liver inflammation and cell death^32^. Growing evidence indicates that the NLRP3 inflammasome has an important role in inflammation and fibrosis in NASH^36^. Of note, NLRP3 inflammasome components have been found to be markedly increased in the liver of NASH patients and also in experimental models of NASH^20^. In addition, recent work by Wree *et al*. showed that constitutive expression of the active form of NLRP3 inflammasome is associated to extensive liver inflammation, hepatocyte cell death by pyroptosis and liver fibrosis in the same mouse model used by us^27^. In the present study we observed that ANDRO clearly reduced the expression of ASC and NLPR3 in CDAA-fed mice as well as decreased hepatic mRNA levels of CASP1 and IL-1β in this setting. Thus, ANDRO’s modulatory effect on the inflammasome seems to be a novel effect of this botanical compound that deserves further study. Interestingly, two recent animal studies have found that beneficial effects of some natural compounds in experimental NASH are also related to modulation of the inflammasome in the liver. Thus, Vivoli *et al*. found that berberine, also an Ayurvedic medicine as ANDRO, interferes with activation of the NLRP3 inflammasome pathway and markedly improved parameters of liver injury and necro-inflammation in a mouse model of NASH^37^. Also, Yang *et al*.^38^ showed that NLRP3 inhibition by sulforaphane, a compound derived from cruciferous vegehtables, resulted in amelioration of hepatic steatosis in high-fat fed mice^38^. Both results are in agreement with our observations and suggest that reduced HTC, also observed by Ding *et al*.^16^, and liver inflammation in ANDRO-treated mice fed with CDAA diet compared with vehicle-treated animals fed with the same diet may be related, at least in part, to modulation of hepatic inflammasome. Of note, since the CDAA diet feeding model is of more intense and long-term model of NASH, with all liver features of the disease compared to more ‘metabolic’ models such as high-fat diet fed mice or short-term nutritional models such as methionine-choline-deficient diet-induced NASH, we were able to also document an anti-fibrotic action of ANDRO in the setting of NASH. Thus, ANDRO modulatory effect on the inflammasome seems to be a novel effect of this botanical compound that deserves further study.

Given the beneficial effects of ANDRO in our model of NASH we think that, after further studies replicating our findings, this compound could be tested in pilot clinical studies. Of note, ANDRO and ANDRO-related compounds have been tested in humans in short-term clinical trials in patients with rheumatoid arthritis^39^ and inflammatory bowel disease with no relevant safety issues^40^. Some potential limitations for the clinical use of ANDRO are related to its high insolubility, poor bioavailability, and short plasma half-life although efforts are being made to improve these shortcomings through modifying the molecule^41^ or complexing it with bigger compounds such as polyethyleneglycol^42^.

In summary, ANDRO administration attenuates liver damage in NASH reducing inflammation and fibrosis adding to the existent evidence of hepatoprotective effects of this botanical compound. Future work should focus on confirmation of ANDRO’s effect in other NAFLD/NASH models, and the design of pilot clinical trials with ANDRO for patients with this increasingly important disease.

## Electronic supplementary material

Supplementary Information
