# Supplementary material for: Andrographolide Ameliorates Inflammation and Fibrogenesis and Attenuates Inflammasome Activation in Experimental Non-Alcoholic Steatohepatitis
Source: Sci Rep. 2017 Jun 14;7:3491. doi: 10.1038/s41598-017-03675-z (PMC5471224; doi:10.1038/s41598-017-03675-z)
Supplement: Supplementary file 1 — Supplementary Information [file 41598_2017_3675_MOESM1_ESM.pdf]

SUPPLEMENTARY MATERIAL FOR:

SREP-16-37389B

ANDROGRAPHOLIDE AMELIORATES INFLAMMATION AND FIBROGENESIS AND ATTENUATES INFLAMMASOME ACTIVATION IN EXPERIMENTAL NON-ALCOHOLIC STEATOHEPATITIS

Daniel Cabrera[1,2], Alexander Wree[3], Davide Povero[3], Nancy Solís[1], Alejandra Hernandez[1], Margarita Pizarro[1], Han Moshage[4], Javiera Torres[5], Ariel E. Feldstein[3], Claudio Cabello-Verrugio[6,7], Enrique Brandan[8], Francisco Barrera[1], Juan Pablo Arab[1,9] and Marco Arrese[1,10]*

Corresponding author:

Marco Arrese, M.D.
Departamento de Gastroenterología
Escuela de Medicina
Pontificia Universidad Católica de Chile, Marcoleta #367, Santiago, ChilePostal code 6510260, Phone: 56-2-23543820, Fax: 56-2-26397780, e-mail: marrese@med.puc.cl

Supplementary Table 1. Oligonucleotide primers and probes used for Real time-PCR			
**mRNA**	**Forward 5’- 3’**	**Reverse 5’-3’**	**speci**
18S	CGGCTACCACATCCAAGGA	CCAATTACAGGGCCTCGAAA	Rattus Norvegicus
18S	GCCGCTAGAGGTGAAATTCT	TCGGAACTACGACGGTATCT	Homo Sapiens
Arg1	CTC CAA GCC AAA GTC CTT AGA G	AGG AGC TGT CAT TAG GGA CAT C	Mus Musculus
ASC	CTT GTC AGG GGATGA ACT CAA AA	GCC ATA CGA CTC CAG ATA GTA GC	Mus Musculus
Casp1	ACA AGG CAC GGG ACC TAT G	TCC CAG TCA GTC CTG GAA ATG	Mus Musculus
COL1A1	GCT CCT CTT AGG GGC CAC T	CCA CGT CTC ACC ATT GGG G	Mus Musculus
COL1A1	TGGTGAACGTGGTGTACAAGGT	CAGTATCACCCTTGGCACCAT	Rattus Norvegicus
CTGF	GGG CCT CTT CTG CGA TTT C	ATC CAG GCA AGT GCA TTG GTA	Mus Musculus
F4/80	TGA CTC ACC TTG TGG TCC TAA	CTT CCC AGA ATC CAG TCT TTC C	Mus Musculus
IL-1b	TGGCGAGCTCAGGTACTTCT	GCTCTGGGATTCTCTTCAGCC	Homo Sapiens
iNOS	GTT CTC AGC CCA ACA ATA CAA GA	GTG GAC GGG TCG ATG TCA C	Mus Musculus
MCP1	TTA AAA ACC TGG ATC GGA ACC AA	GCA TTA GCT TCA GAT TTA CGG GT	Mus Musculus
MMP2	CAA GTT CCC CGG CGA TGT C	TTC TGG TCA AGG TCA CCT GTC	Mus Musculus
pro-IL-1β	GAA ATG CCA CCT TTT GAC AGT G	CTG GAT GCT CTC ATC AGG ACA	Mus Musculus
TGF-β	GGGCTACCATGCCAACTTCTG	GAGGGCAAGGACCTTGCTGTA	Rattus Norvegicus
TIMP-1	CTT GGT TCC CTG GCG TAC TC	ACC TGA TCC GTC CAC AAA CAG	Mus Musculus
TNF-α	CCC TCA CAC TCA GAT CAT CTT CT	GCT ACG ACG TGG GCT ACA G	Mus Musculus
α-SMA	GTC CCA GAC ATC AGG GAG TAA	TCG GAT ACT TCA GCG TCA GGA	Mus Musculus
α-SMA	GCCAGTCGCCATCAGGAAC	CACACCAGAGCTGTGCTGTCTT	Rattus Norvegicus
